# PFP-LHCINCA: Pyramidal Fixed-Size Patch-Based Feature Extraction and Chi-Square Iterative Neighborhood Component Analysis for Automated Fetal Sex Classification on Ultrasound Images

**DOI:** 10.1155/2022/6034971

**Published:** 2022-05-18

**Authors:** Ela Kaplan, Tekin Ekinci, Selcuk Kaplan, Prabal Datta Barua, Sengul Dogan, Turker Tuncer, Ru-San Tan, N Arunkumar, U. Rajendra Acharya

**Affiliations:** ^1^Department of Radiology, Adıyaman Training and Research Hospital, Adiyaman 1164, Turkey; ^2^Department of Obstetrics and Gynecology, Malatya Turgut Ozal University Training and Research Hospital, Malatya 44330, Turkey; ^3^Department of Obstetrics and Gynecology, Adıyaman Gozde Hospital, Adiyaman 1164, Turkey; ^4^School of Business (Information System), University of Southern Queensland, Toowoomba, QLD 4350, Australia; ^5^Faculty of Engineering and Information Technology, University of Technology Sydney, Sydney, NSW 2007, Australia; ^6^Department of Digital Forensics Engineering, College of Technology, Firat University, Elazig 23119, Turkey; ^7^Department of Cardiology, National Heart Centre Singapore, Bukit 169609, Singapore; ^8^Duke-NUS Medical School, Bukit 169857, Singapore; ^9^Rathinam College of Engineering, Coimbatore, India; ^10^Ngee Ann Polytechnic, Department of Electronics and Computer Engineering, Bukit 599489, Singapore; ^11^Department of Biomedical Engineering, School of Science and Technology, SUSS University, Bukit 599491, Singapore; ^12^Department of Biomedical Informatics and Medical Engineering, Asia University, Taichung 180-8629, Taiwan

## Abstract

**Objectives:**

Fetal sex determination with ultrasound (US) examination is indicated in pregnancies at risk of X-linked genetic disorders or ambiguous genitalia. However, misdiagnoses often arise due to operator inexperience and technical difficulties while acquiring diagnostic images. We aimed to develop an efficient automated US-based fetal sex classification model that can facilitate efficient screening and reduce misclassification.

**Methods:**

We have developed a novel feature engineering model termed PFP-LHCINCA that employs pyramidal fixed-size patch generation with average pooling-based image decomposition, handcrafted feature extraction based on local phase quantization (LPQ), and histogram of oriented gradients (HOG) to extract directional and textural features and used Chi-square iterative neighborhood component analysis feature selection (CINCA), which iteratively selects the most informative feature vector for each image that minimizes calculated feature parameter-derived k-nearest neighbor-based misclassification rates. The model was trained and tested on a sizeable expert-labeled dataset comprising 339 males' and 332 females' fetal US images. One transverse fetal US image per subject zoomed to the genital area and standardized to 256 × 256 size was used for analysis. Fetal sex was annotated by experts on US images and confirmed postnatally.

**Results:**

Standard model performance metrics were compared using five shallow classifiers—k-nearest neighbor (kNN), decision tree, naïve Bayes, linear discriminant, and support vector machine (SVM)—with the hyperparameters tuned using a Bayesian optimizer. The PFP-LHCINCA model achieved a sex classification accuracy of ≥88% with all five classifiers and the best accuracy rates (>98%) with kNN and SVM classifiers.

**Conclusions:**

US-based fetal sex classification is feasible and accurate using the presented PFP-LHCINCA model. The salutary results support its clinical use for fetal US image screening for sex classification. The model architecture can be modified into deep learning models for training larger datasets.

## 1. Introduction

Noninvasive fetal sex determination is feasible with maternal transabdominal ultrasound (US) examination from about the 12^th^ week of gestation and becomes more reliable as fetal sex organs mature. It is clinically indicated in pregnancies at risk of X-linked genetic disorders or ambiguous genitalia. Early sex classification has important implications for the consideration of termination and hormone therapy to drive differential sexual maturation. Manually assessed upward and downward directions of the genital tubercle on the fetal US are the earliest signs of the male and female sex, respectively, that are best confirmed by later definitive development of the phallus and labia [1–3]. Misdiagnoses often arise due to malformed external genitalia, operator inexperience and/or fatigue, and unsuccessful attempts at obtaining diagnostic images of the moving fetus that may change its position variably within the uterus (which is somewhat mitigated on three-dimensional ultrasound (US) imaging) [4, 5]. These morphological, human, and technical factors justify the need for and drive the development of the automated computer-aided classification of fetal biophysical signals that can be incorporated into efficient high-throughput fetal sex screening to reduce misclassification [6, 7]. Khanmohammadi et al. [6] reported a deep learning model that attained 91.00% and 93.00% accuracies for fetal sex determination with leave-one-out cross-validation and hold-out method, respectively, on a dataset comprising 1000 phonocardiogram signals from the Shiraz University Fetal Heart Sounds Database. Maysanjaya et al. [7] applied learning vector quantization to 89 fetal US images and attained a 0.05 learning rate but modest 63.00% accuracy for sex classification. Aljuboori et al. [8] used a novel hybrid filter and fuzzy C-mean based method to extract and select features on 100 fetal US images to separate them into male and female clusters, attaining superior 94.00% and 90.00% accuracies, respectively. Of note, both US studies were based on small datasets, which limits the generalizability of the findings.

The novel sides of this model are highlighted below:

- A new fetus image dataset was collected to diagnose the gender of the fetus.
- Pooling functions are simple decomposition models, and they have routing problem in machine learning. A simple solution has been used to overcome/solve this problem, and it is named multiple average pooling.
- A hybrid feature extractor has been presented in this work. The proposed feature extractor generates both shape and texture features from an image.
- A new feature selector has been proposed, and this feature selector is named CINCA since two feature selection functions have been used together: Chi2 and iterative NCA.
- The classification results have been calculated using five optimized classifiers to give a benchmark about this dataset and our model.
- A new hand-modeled learning architecture has been proposed to get high classification results with low time complexity.

Contributions of our proposal are as follows:

- In the literature, there are variable deep and handcrafted models to achieve high classification performance. In this work, a new generation patch-based handcrafted features-based image classification model has been proposed. The main objective of this model is to use the effectiveness of exemplar models like ViT and MLP-Mixer.
- Our proposal (fixed-size patch-based gender classification) contains feature extraction, feature selection, and classification phases. Each phase has individual novelties. In medical images, local abnormalities are very valuable to extract important/valuable information. Therefore, fixed-size patch-based local feature generation has been used. Herein, a hybrid handcrafted feature extractor has been used. This feature extractor can create both textural and shape features using LPQ and HOG together. To remove the redundant features, a hybrid and iterative feature selector has been proposed. By using Chi2, the length of the features has been decreased in a short time, and INCA chooses the best features. To classify these selected features, a shallow classifier has been used, and the parameters of this shallow classifier have been tuned using Bayesian optimization. This model reached 99.11% classification accuracy using a kNN classifier with tenfold cross-validation. In view of this, we have proposed a cognitive architecture since each phase has been dedicated to attaining high classification performance with a low time burden.

## 2. Materials and Methods

**Figure 1:**
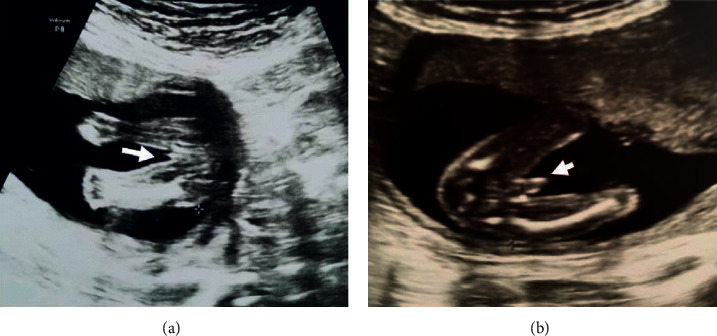
Examples of fetal ultrasound images showing transverse views of a 20-week female fetus (a) and an 18-week male fetus (b) demonstrating labial folds and phallus, respectively (arrowheads).

We performed a retrospective analysis of fetal US images acquired from 671 pregnant women who underwent routine second trimester US scans at the Adıyaman Maternity and Children's Hospital in Malatya, Turkey, between January 2021 and October 2021. Example images are shown in Figure 1. Local ethics committee approval had been obtained for the study, which was performed in accordance with the Declaration of Helsinki.

Table 1 summarizes the demographic and clinical information of the analyzed subjects.

All transabdominal US studies were performed using a Voluson P8 scanner (GE Healthcare, Milwaukee, Wisconsin, USA) by a radiologist with five years' fetal US experience (EK). From the initial midsagittal plane with the fetus in the neutral position, the probe was rotated to obtain the transverse view of the fetal external genitalia (Figure 1), which was zoomed in and enlarged. On the midsagittal view, upward and downward directions of the genital tubercles are the earliest signs of male and female fetuses, respectively [4]. A more mature male fetus would typically display a uniform dome-shaped structure representing the fetal penis and fetal scrotum as well as a longitudinal midline echogenic line at the base of the fetal penis [4], while a more mature female fetus would typically display two or four parallel lines representing the labia major and minor, which are best seen in the transverse plane [4]. In this study, zoomed transverse images of the genital area (*n* = 671, one image per subject) were stored in JPEG format for analysis and counterchecked by an obstetrician with ten years' experience (SK). After birth, each neonate's sex was confirmed from information on the birth certificate. In total, there were 339 male and 332 female fetuses/neonates in the study. The number of images used is tabulated in Table 2.

**Figure 2:**
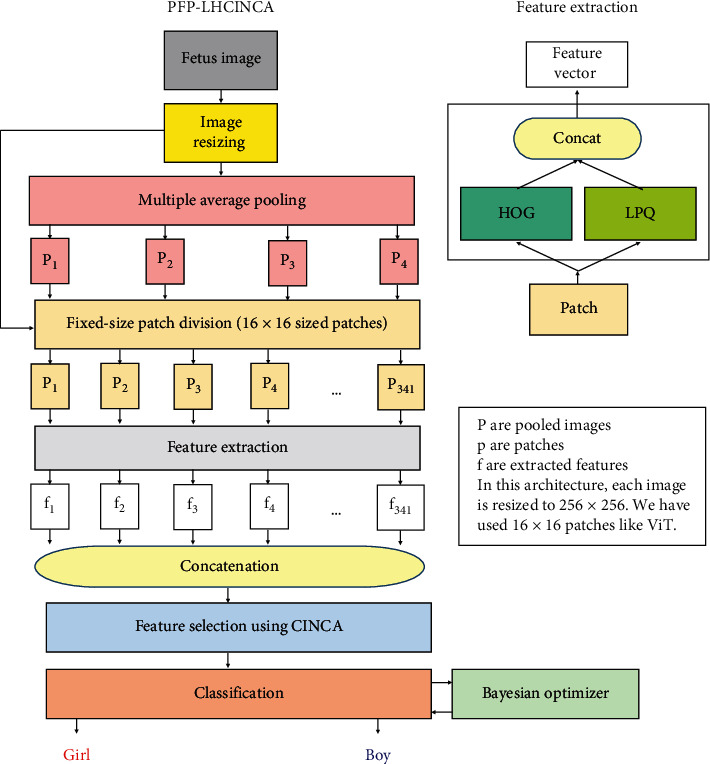
Schematic of the PFP-LHCINCA fetal ultrasound image sex classification model.

We aim to develop an effective feature engineering model with an efficient handcrafted feature extraction architecture that at the same time incorporates elements of deep learning. A new hybrid feature selector, CINCA, is proposed to select the most discriminative/meaningful feature vectors to be fed to standard shallow classifiers. Distinct phases of our PFP-LHCINCA model—feature extraction, feature selection, and classification—are detailed in the text below and illustrated in Figure 2.

The purpose is to extract comprehensive multilevel features. First, all images are resized to 256 × 256 resolution. Next, we apply fixed-size patch separation, a technique that has been used in works like vision transformers [9], as well as global average pooling, which mimics convolutional neural networks [10], to create a multilevel feature extraction model. Each image is divided to construct a pyramid of 2 × 2 (four), 4 × 4 (16), 8 × 8 (64), and 16 × 16 (256) nonoverlapping blocks or pooled images with decreasing size: 128 × 128, 64 × 64, 32 × 32, and 16 × 16, respectively (P1, P2, P3, and P4 in Figure 2). The original 256 × 256 images and all pooled images are each decomposed into 16 × 16 images, i.e., a fixed-size patch, using average pooling. By dividing the original 256 × 256 images and all pooled images into 16 × 16 sized patches, the total number obtained from this fixed-size division is 341 (= 256 + 64+16 + 4 + 1). LPQ [11, 12] and HOG [13] are then applied to each of the 341 16 × 16 fixed-size patches to generate both directional and textural features (256 LPQ and 36 HOG features), which results in 341 feature vectors with a length of 292 (= 256 + 36) each that are all concatenated to form a new matrix to be input to the feature selector.

The pseudocode of the presented fetus image classification model is given in Algorithm 1.

The pseudocode of this model is demonstrated in Algorithm 1. The details about the used methods in our hand-modeled architecture are given below.

HOG [13] is one of the widely used image descriptors in the literature and has been used for human detection problems. It is a histogram-based feature extraction function. Directions (angles) and gradients (magnitude) are used to create a feature vector. Gradients have been calculated by using Sobel kernels (it has been used for edge detection). It is a very successful feature extractor for shape detection.

(1) $$\begin{array}{c} m=\sqrt{G_{x}^{2}+G_{y}^{2}},\\ \alpha =\arctan\left(\frac{G_{x}}{G_{y}}\right). \end{array}$$

Herein, *m* is the magnitude, *α* is the angel of gradients (directions), and  *G*_*x*_ and *G*_*y*_ are horizontal and vertical gradients.

LPQ [11, 12] is a commonly used textural feature extractor, and it is a local binary pattern (LBP) like an image descriptor. The image is divided into *N* × *M* sized overlapping blocks in this method. Fourier transform and blurring methods have been used to generate effective textural features.

HOG extracts shape-based features, and LPQ is a commonly known/preferred textural feature extraction function and generates textural features at space and frequency domains. By using both of them, shape and texture features have been generated. Herein, the fundamental purpose of the used hybrid (HOG + LPQ) features extractor is to generate both textural and directional features.

The generated directional (using HOG) and textural (using LPQ) features are fed as input of Chi-square iterative neighborhood component analysis (CINCA). The description of the CINCA selector is given in the next section.

We designed an efficient hybrid feature selection function that incorporates Chi2 [14] and neighborhood component analysis [15], which effectively selects features with the minimum classification errors using weighted k-nearest neighbor (kNN) [16]. The main objective of the presented CINCA selector is to decrease the time complexity of the huge feature vector since the Chi2 selector is one of the fastest (quick response) feature selection functions in the literature [14]. First, Chi2 is applied in the first layer to filter the top most valuable 1000 extracted features out of 99,572 (= 341 × 292) extracted features, which decreases the time burden/complexity of downstream iterative feature selection processes considerably. As neither Chi2 nor NCA per se can execute beyond a single feature selection step, the 1000 selected features are fed to the second layer composed of the INCA selector [17], which uses feature parameters to calculate kNN misclassification rates to iteratively narrow the selection to the optimal/best feature vector with the lowest misclassification rate automatically using tenfold cross-validation. Based on the novel hybrid feature selection function CINCA, the most informative feature vector in our experiment is the one with 498 features.

The final classification phase is the simplest in our model. Five standard shallow classifiers—kNN, linear discriminant (LD), naïve Bayes (NB), support vector machine (SVM), and decision tree (DT)—are chosen, and their respective hyperparameters tuned using a Bayesian optimizer [18]. The results of the PFP-LHINCA model with five classifiers are then compared.

A stepwise mathematical account of our PFP-LHCINCA model is given below and implemented in the MATLAB 2021b environment (the parameter settings are summarized in Table 2).

**Step 1 .**

Load the fetus US image dataset, and read each US image.

**Step 2 .**

Apply image resizing to each image to set a size of 256 × 256.

**Step 3 .**

Decompose the image using four levels of average pooling (multilevel decomposition) as given below.

(2) Pi=avpIm,2i×2i, i∈1,2,3,4.

Herein, 2 × 2, 4 × 4, 8 × 8, and 16 × 16 sized nonoverlapping blocks are used to create decomposed images (*P*) using average pooling (avp(., .)). Im is the used fetal US image.

**Step 4 .**

Divide image (Im) and pooled images (*P*) into 16 × 16-sized patches.

(3) pk=Imt:t+15,r:r+15, t∈1,17,…,241, r∈1,17,…,241,k∈1,2,…,256,

(4) $$\begin{array}{c} p_{h+256}=P_{1}\left(t:t+15,r:r+15\right),\;\\ t\in \left\{1,17,\ldots ,113\right\},\;\\ r\in \left\{1,17,\ldots ,113\right\},\\ h\in \left\{1,2,\ldots ,64\right\},\\ t\in \left\{1,17,\ldots ,49\right\},\\ \;r\in \left\{1,17,\ldots ,49\right\},\\ v\in \left\{1,2,\ldots ,16\right\},\\ t\in \left\{1,17\right\},\;\\ r\in \left\{1,17\right\},\\ v\in \left\{1,2,\ldots ,4\right\}, \end{array}$$

(5) $$\begin{array}{c} p_{v+320}=P_{2}\left(t:t+15,r:r+15\right),\;\\ t\in \left\{1,17,\ldots ,49\right\},\\ \;r\in \left\{1,17,\ldots ,49\right\},\\ v\in \left\{1,2,\ldots ,16\right\},\\ t\in \left\{1,17\right\},\;\\ r\in \left\{1,17\right\},\\ v\in \left\{1,2,\ldots ,4\right\}, \end{array}$$

(6) $$\begin{array}{c} p_{c+336}=P_{3}\left(t:t+15,r:r+15\right),\;\\ t\in \left\{1,17\right\},\;\\ r\in \left\{1,17\right\},\\ v\in \left\{1,2,\ldots ,4\right\}, \end{array}$$

(7) $$\begin{array}{c} p_{341}=P_{4}. \end{array}$$

In (4) to (8), the patch division process is defined, and 341 patches are obtained.

**Step 5 .**

Extract features from each patch.

(8) $$\begin{array}{c} f_{j}=\text{concat}\left(\text{LPQ}\left(p_{j}\right),\text{HOG}\left(p_{i}\right)\right),\;\;j\in \left\{1,2,\ldots ,341\right\}, \end{array}$$

where concat(.) is a concatenation function. Herein, 341 feature vectors are extracted by deploying LPQ and HOG feature extraction functions together.

**Step 6 .**

Merge the created feature vector to obtain the final feature vector.

(9) $$\begin{array}{c} X=\text{concat}\left(f_{1},f_{2},\ldots ,f_{341}\right), \end{array}$$

where *X* is the merged final feature vector.

**Step 7 .**

Apply Chi2 feature selector to *X*, and calculate qualified indices of *X*.

**Step 8 .**

Choose the most informative 1000 features from *X* deploying the generated indices.

**Step 9 .**

Apply INCA to the 1000 features selected by Chi2. INCA uses parameters (kNN is deployed as a misclassification rate calculator, and the range of INCA is set at 100 to 1000) to select the optimal feature vector with the lowest misclassification rate.

Steps 7–9 constitute the proposed CINCA feature selection function.

**Step 10 .**

Optimize hyperparameters of the used DT [19], LD [20], NB [21], kNN [16], and SVM [22, 23] classifiers by deploying Bayesian optimizer. Herein, 10-fold cross-validation has been utilized as a validation model.

## 3. Results and Discussion

In this work/research, we used MATLAB 2021b programming environment to realize our proposal. We used a simple configured laptop, and this laptop has i7-7700 central processing unit (CPU), 16 GB memory, and 256 GB solid-state hard disk with Windows 10.1 ultimate operating system. We used functions to create this hand-modeled image classification model. The used functions were stored as *m* files. Our model is a parametric model, and our used parameters are tabulated in Table 3.

To compare the performance of the PFP-LHCINCA using the five different classifiers, standard performance metrics—precision, recall, accuracy, and F1 score [24, 25]—as well as the confusion matrices of every classifier are presented. The mathematical definitions of these performance metrics are given in the following equations:

(10) $$\begin{array}{c} \text{accuracy}=\frac{tp+tn}{tp+tn+fp+fn}, \end{array}$$

(11) $$\begin{array}{c} \text{precision}=\frac{tp}{tp+fp}, \end{array}$$

(12) $$\begin{array}{c} \text{recall}=\frac{tp}{tp+fn}, \end{array}$$

(13) $$\begin{array}{c} \text{F}1=2\frac{\text{recall}\times \text{precision}}{\text{recall}+\text{precision}}. \end{array}$$

Herein, *tp*,  *tn*,  *fp*, and *fn* are the numbers of true positives, true negatives, false positives, and false negatives, respectively.

**Figure 3:**
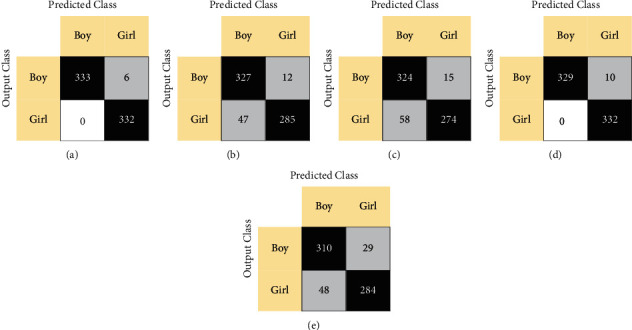
Confusion matrices of PFP-LHCINCA model by classifier type. kNN: k-nearest neighbor; LD: linear discriminant; NB: naïve Bayes; SVM: support vector machine; DT: decision tree. (a) kNN. (b) LD. (c) NB. (d) SVM. (e) DT.

**Figure 4:**
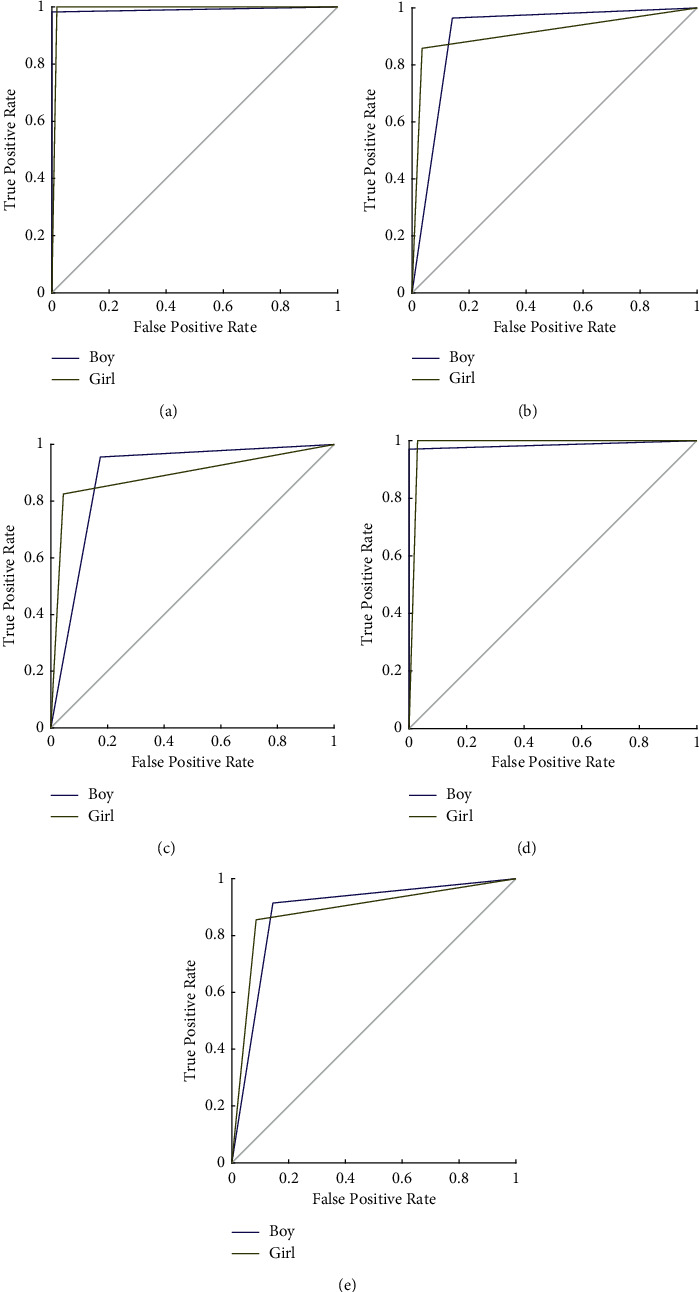
Receiver operating characteristic curves of the classifiers used in the model. AUC: area under curve; kNN: k-nearest neighbor; LD: linear discriminant; NB: naïve Bayes; SVM: support vector machine; DT: decision tree. (a) kNN, 0.99, (b) LD. (c) NB, 0.89. (d) SVM, 0.98. (e) DT, 0.88.

Confusion matrices and receiver operating characteristic (ROC) curves comparing model performance with each classifier are presented in Figures 3 and 4, respectively. Furthermore, Table 4 indicates the overall classification results of the proposed PFP-LHCINCA model.

**Figure 5:**
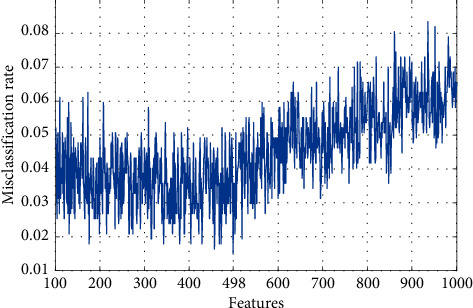
Determining the optimal feature vector with the lowest misclassification using an iterative neighborhood component analysis.

This study observed good diagnostic performance for our proposed PFP-LHCINCA model using a large fetal US image dataset comprising 339 male and 332 female fetuses. In the PFP-LHCINCA model, handcrafted feature engineering involving both fixed-size patch and pyramidal feature generation architectures is used to generate 341 16 × 16 fixed-size patches per standardized resized 256 × 256 zoomed image of the fetal genital area. LPG and HOG extracted 292 features from each patch, i.e., 99,572 features per image. These extracted features are merged into a new matrix which is then fed to a new hybrid feature selection function CINCA, which incorporates Chi2 to filter the top 1000 extracted features and INCA to narrow the selection to the most informative feature vector using iterative calculations of the feature parameter-derived kNN misclassification rates. The optimal feature vector possesses 498 features with minimum misclassification (0.0149), which translates into 98.51% accuracy using the kNN classifier (see Figure 5).

The optimal feature vectors from all image samples are input into five standard shallow classifiers, including kNN, with corresponding hyperparameters tuned using a Bayesian optimizer. The best-performing kNN classification accuracy improved from 98.51% (unadjusted) to 99.11% by tuning the hyperparameters. In addition, our model has performed well compared with other published methods for US-based fetal sex classification (see Table 5).

As can be seen from Table 5, the proposed PFP-LHCINCA attained over 99% classification accuracy using simple methods together. Moreover, our proposal outperforms the other methods.

Many works have been carried out to evaluate the correct determination of fetal gender by ultrasonography in pregnancy, and ultrasonographic examination is still the most effective and accurate method. However, cases where parallel lines cannot be clearly evaluated in the female gender can be confused with parallel lines located in the scaling midline, or incorrect gender determination can be made by mixing the umbilical cord with the penis, which is quite common. The most important reasons for this are the more time spent on the evaluation of organs and fetal development in detailed ultrasonographic screening or pregnancy examination, the fetal mobility, or the inability to obtain the desired fetal position. Detection of fetal sex with artificial intelligence methods with such high accuracy rates can be beneficial for both radiologists and obstetricians, and it can be very helpful in responding correctly to parental demands by eliminating bias and facilitating objective evaluation.

The highlights of this research are as follows:

- A new US-based fetus sex classification model, PFP-LHCINCA, that has attained excellent performance using handcrafted features is presented.
- The model employed a novel feature extraction architecture that deployed fixed-size patch division and average pooling, combined with shallow feature extractors LPQ and HOG to generate comprehensive multilevel features efficiently.
- The model accuracy rates are 88% or better when combined with five standard shallow classifiers with hyperparameters tuned using a Bayesian optimizer. Best accuracy rates are obtained with SVM and kNN classifiers (>98%).
- While we have used 16 × 16 fixed-size patches (inspired by vision transformers [9]) with four decomposition levels using average pooling, these parameters are modifiable. In this respect, the proposed architecture is an extendable computer vision model.
- A hybrid feature selection function, CINCA, combining Chi2 and INCA, has been presented; it automatically selected the most informative feature vector with the lowest kNN misclassification rate derived from input feature parameters.
- As the feature extraction phase has adapted elements inspired by deep learning models, i.e., fixed-size patch division and average pooling-based image decomposition, and the feature selection and classification phases can execute autonomously, the model architecture can be modified into new deep learning models for training larger datasets.

In the real-world applications, we can propose a new intelligent application to detect genders. Our proposed method can be applied to a big image dataset. All phases will be implemented in the training phase, and the optimized parameters will be obtained. In the testing phase, only feature extraction phase will be implemented to generate features of the testing observation. By using the calculated indices in the training phase, the features are selected, and by using the optimized parameters, classification will be conducted.

## 4. Conclusions

An automated US-based fetal sex classification method that has been trained and tested on a new large US image dataset is presented in this work. The novel image classification architecture deployed fixed-size patch division and average pooling-based image decomposition. This model created 341 patches, and handcrafted features were extracted from each patch using LPQ and HOG descriptors together. A hybrid CINCA function chooses the most valuable feature vectors, and the classification ability of these features is tested using five shallow classifiers. Hyperparameters of these classifiers are tuned using Bayesian optimization. The optimized kNN classifier, SVM, LD, NB, and DT attained classification accuracy of 99.11%, 98.51%, 91.21%, 89.12%, and 88.52%, respectively. Moreover, the AUC values of these classifiers on ROC analyses range from 0.88 to 0.99. SVM and kNN classifiers also attain 100% recall for detecting female fetuses. These salutary results demonstrate the feasibility and accuracy of our proposed PFP-LHCINCA model, which support its use for fetal sex classification with US images in the clinic.

We plan to acquire a larger fetal US dataset, which will allow training and testing using deep models. By training a deep learning network on the extensive ultrasound dataset, the calculated weights can be used to transfer learning and develop a new fully automated fetal sex classification application that can provide real-time online triage and alert when embedded directly on ultrasound imaging devices. Moreover, our other future intention is to develop an intelligent assistant to help operator doctors in operations.

## Figures and Tables

**Algorithm 1:**
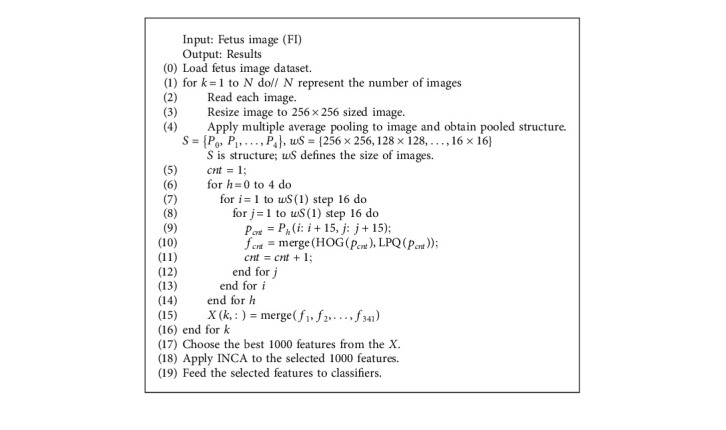
Algorithm 1 Pseudocode of our proposal.

**Table 1:** Temperature and wildlife count in the three areas covered by the study.

Age (years), mean ± SD	25.17 ± 13.72
Obstetric data	
Gravida, mean ± SD	2.3 ± 0.7
Parity, mean ± SD	2.1 ± 1.2
Abortus, mean ± SD	0.9 ± 0.6

Vaginal birth, *n* (%)	553 (82.4)

Caesarean section, *n* (%)	118 (17.6)

**Table 2:** Number of images used.

Male	339
Female	332
Total	671

**Table 3:** MATLAB implementation and parameter settings of the PFP-LHCINCA model.

Method	Parameter
Image resizing	256 × 256
Image decomposition	Average pooling with four levels using 2 × 2, 4 × 4, 8 × 8, and 16 × 16
Patch division	16 × 16 sized patches
LPQ and HOG feature extraction	341 (256 LPQ and 36 HOG) features are extracted for each patch
Feature merging	The concatenation function is merged
Chi2	The most informative 1000 features are selected
INCA	Range: [100, 1000]; error function: kNN with 10-fold CV. Herein, *k* is 1, the distance metric is Euclidean, and weight is none
Classifiers	kNN: *k* = 70, distance: correlation, weight: squared inverse
	LD: discriminant type: linear, gamma: 0
	NB: kernel: normal, support: unbounded
	SVM: kernel function: Gaussian, box constraint: 3, kernel scale: 5.6
	DT: split criterion: deviance, maximum number of splits: 51, surrogate: off
Bayesian optimizer	Acquisition function: expected improvement per second plus, iterations: 100

**Table 4:** Performance metrics of PFP-LHCINCA model by classifier type.

Classifier	Accuracy (%)	Precision (%)	Recall (%)	F1 score (%)
kNN	99.11	99.11	99.12	99.11
LD	91.21	91.70	91.15	91.43
NB	89.12	89.81	89.05	89.43
SVM	98.51	98.54	98.53	98.53
DT	88.52	88.66	88.49	88.58

kNN: k-nearest neighbor; LD: linear discriminant; NB: naïve Bayes; SVM: support vector machine; DT: decision tree.

**Table 5:** Comparison of PFP-LHCINCA model with published results of other ultrasound-based fetal sex classification methods.

Study	Method	Dataset	Best accuracy (%)
Maysanjaya et al. [7]	Learning vector quantization, artificial vector quantization	64 males 25 females	63.0%

Aljuboori et al. [8]	Fuzzy C-mean, discrete wavelet transform, local binary pattern, median, Laplacian filters	50 males 50 females	94.0%

PFP-LHCINCA	Pyramidal fixed-size patch division, local phase quantization and histogram of oriented gradients based feature extraction, hybrid Chi2 and iterative neighborhood component analysis feature selection	339 males 332 females	99.11% (kNN classifier tuned with Bayesian optimizer)

## Data Availability

The data presented in this study are available on request from the corresponding author. The data are not publicly available due to the Ethical Committee Institution restrictions.
